# Monitoring Wood Degradation during Weathering by Cellulose Crystallinity

**DOI:** 10.3390/ma5101910

**Published:** 2012-10-19

**Authors:** Francesca Lionetto, Roberta Del Sole, Donato Cannoletta, Giuseppe Vasapollo, Alfonso Maffezzoli

**Affiliations:** Department of Engineering for Innovation, University of Salento, via Monteroni, Lecce 73100, Italy; E-Mails: roberta.delsole@unisalento.it (R.D.S.); donato.cannoletta@unisalento.it (D.C.); giuseppe.vasapollo@unisalento.it (G.V.); alfonso.maffezzoli@unisalento.it (A.M.)

**Keywords:** crystallinity, pine wood, X-ray diffraction (XRD), Fourier transform infrared (FT-IR), outdoor weathering, wood coatings, lignin, cellulose, wood degradation

## Abstract

The degree of crystallinity of cellulose was used for assessing the degradation level of coated and uncoated samples of pine wood after weathering. X-ray diffraction (XRD) and Fourier Transform Infrared (FT-IR) spectroscopy measured the changes in the surface crystallinity of cellulose resulting from weathering, both natural and artificial. Both techniques revealed an increase in the crystallinity index (CI) of cellulose when wood was subjected to weathering. An increase in the size of crystallites was also observed by XRD measurements. These results were related to the reduction of the amorphous fractions of wood, and, consequently, to the enrichment of the relative crystalline content. Thanks to FT-IR analysis, the degradation of hemicellulose was observed for uncoated samples after exposure to artificial weathering. The effect of weathering was less evident on coated samples because of the protective action of the coating. A good correlation between the crystallinity indexes obtained from FT-IR and XRD was found. The experimental results proved that the proposed method may be a very useful tool for a rapid and accurate estimation of the degradation level of wood exposed to weathering. This methodology can find application in the field of conservation and restoration of wooden objects or in the industry of wood coatings.

## 1. Introduction

During outdoor exposure, wood can undergo severe changes of its physical and structural properties due to the combined effect of sunlight, oxygen, moisture, atmospheric pollutants and micro-organisms. The combination of oxygen and solar radiation rapidly induces oxidation of lignin and hemicellulose and depolymerisation of cellulose. Many of the reaction products are water-soluble so they are easily removed from the wood surface by rain, leading to a material loss and a roughened and discoloured surface [1,2].

Despite the use of coatings to prevent weathering, degradation of wood beneath the coating still occurs, albeit at a reduced rate [3,4]. Generally, the degradation of wood has been studied by weight loss measurements, colour measurements, mechanical tests, FT-IR spectroscopy, optical and electron microscopy [1,5,6,7,8].

Wood is mainly composed of cellulose, hemicellulose and lignin and can be analysed by a combination of characterisation techniques commonly used in the polymer science [9,10,11]. Wood crystallinity may be a suitable tool for estimating the level of wood degradation during weathering [12]. As is well known, the crystalline fraction of wood is given only by cellulose, since the other two main wood components, hemicellulose and lignin, are amorphous. The crystallinity of wood has an important effect on the physical, mechanical and chemical properties of wood-based materials. For example, Young’s modulus, tensile strength, dimensional stability, density and hardness increase with crystallinity, while moisture regain, dye sorption, chemical reactivity, swelling and flexibility decrease. Therefore, the determination of wood crystallinity may be an approach for understanding the effect of weathering on wood properties.

Unfortunately, the complex chemical composition and texture of wood and its highly anisotropic nature does not make easy the determination of crystallinity. XRD and FT-IR spectroscopy have been used in cellulose and wood studies [13,14,15,16,17,18], to monitor changes in wood crystallinity caused by degradation and biological attack by fungi [19,20]. However, they do not provide reliable absolute values of crystallinity but only relative values [21]. As concerning XRD, in fact, the separation of amorphous background from the diffraction pattern of cellulose crystallites can be affected by significant errors related to their small size (usually between 2.5 and 3.5 nm) [22]. Popescu *et al*. applied FT-IR spectroscopy to provide details about the structural characteristics of hardwood and softwood samples, distinguishing between various kinds of wood [23]. The FT-IR spectroscopic method has also been used to analyse the chemical changes taking place in softwood as a consequence of fungal attack [24] or of environmental exposure [8].

In this work, the relative crystallinity values obtained by XRD and FT-IR spectroscopy on coated and uncoated samples of pine wood (*Pinus sylvestris L*.) after natural and accelerated weathering were compared. The results were analysed with the aim of verifying the correlation between the crystallinity measurements and the extent of wood deterioration resulting from environmental and accelerated weathering. The combined use of these two techniques has enabled the singling out of the level of degradation of lignin and hemicellulose.

## 2. Experimental Section

### 2.1. Sample Preparation

Outdoor weathering tests were made on pine sapwood (*Pinus sylvestris L.*) samples prepared according to the European standard EN 927-3 [25]. The dimensions of wood samples were 200 × 200 × 100 mm^3^ (tangential × radial × longitudinal directions). Half of the wood samples were subjected to a painting cycle, typically applied to window frames, consisting of a primer, intermediate and finishing layers. In all cases, acrylic paints were used, supplied by IVR Chemicals srl with the trade name of XHT22027, XBC20089 and XGC19803, respectively.

Coated and uncoated wood panels were exposed under an angle of 45° facing south at an exposure site in Lecce (Italy) (40.383 N 18.183 E) in a rural environment at a distance of about 20 km from the seaside. Exposure covered a period of 12 months. The values of cumulative annual rainfall and average solar radiation were 441 mm and 135 W/m^2^, respectively.

Artificial weathering was carried out according to the European standard EN 927-6 [26] using a QUV Atlas 65-W Weather-Ometer equipped with a 6500-W xenon-arc UV lamp for 2000 h.

Before the XRD and FT-IR analysis, weathered and unweathered (control) wood samples were conditioned for several weeks at 20 °C and 65% relative humidity to reach a moisture content of 12%.

In the case of coated samples, the coating film was manually removed in several steps to avoid the removal of the first wood layer below the paint. After each removal step, the reduction of coating thickness was carefully checked by an optical microscope. At the end of this procedure, the complete absence of any residue of coating was checked. From the surface immediately below the coating layer, wood powder was extracted and milled to a fine powder (40–80 mesh). For each kind of wood five replicates were tested.

### 2.2. XRD Measurements

Wide angle XRD measurements were carried with a Rigaku Ultima+ diffractometer. The X-ray generator was equipped with a copper tube operating at 40 kV and 26 mA and irradiating the sample with a monochromatic CuKα radiation with a wavelength of 0.154 nm. XRD spectra were acquired at room temperature over the 2θ range of 5°–35° at 0.05° intervals with a measurement time of 1 second per 2θ intervals. For each replicate sample, three spectra were considered.

**Figure 1:**
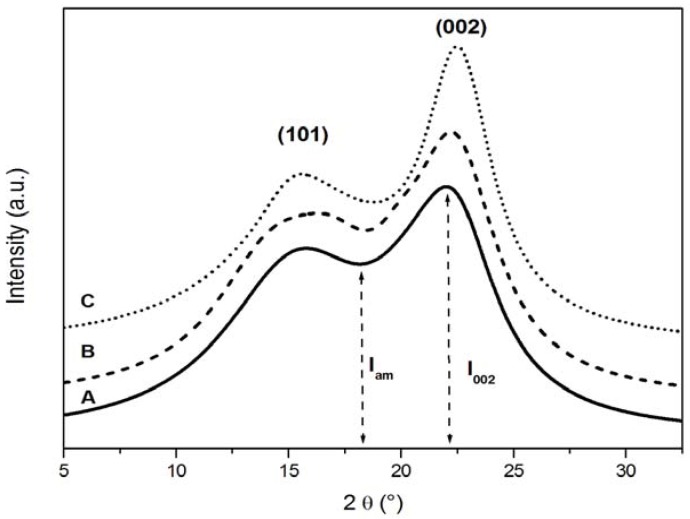
X-ray diffraction (XRD) diffractograms of uncoated wood samples. (**A**) control; (**B**) sample exposed to natural weathering; (**C**) sample artificially weathered.

According to the peak height method developed by Segal *et al.* [27] for native cellulose, the XRD crystallinity index (CI_XRD_) was calculated from the following height ratio:

(1) $$\text{C}{\text{I}}_{\text{XRD}}(\%)=\frac{{\text{I}}_{002}-{\text{I}}_{\text{am}}}{{\text{I}}_{\text{am}}}\text{x}100$$

where I_002_ was the intensity of the 002 crystalline peak at 22° and I_am_ the height of the minimum (I_am_) between the 002 and the 101 peaks, as shown in Figure 1.

The average size of crystallite was calculated from the Scherrer equation with the method based on the width of the diffraction patterns obtained in the X-ray reflected crystalline region. The crystalline size D_002_ was determined by using the diffraction pattern obtained from the 002 lattice planes of cellulose:

(2) $${\text{D}}_{002}=\frac{\text{k}\lambda }{{\text{B}}_{002}\text{cos}\theta }$$

where k is the Scherrer constant (0.84), λ is the X-ray wavelength (0.154 nm), B in radians is the full-width at half of the peak of 002 reflection and θ the corresponding Bragg angle [28].

### 2.3. FT-IR Measurements

Powdered samples were dried for 48 h at 60 °C. FT-IR spectra were obtained on KBr pellets (1 mg of wood powder and 200 mg of KBr, pressed into 13 mm diameter discs at a pressure of 10 Ton). FT-IR spectra were recorded on a JASCO FT-IR 660 plus spectrometer with a resolution of 4 cm^−1^, by 64 scans in the region between 4000 and 650 cm^−1^. For each replicate sample, three spectra were considered.

All spectra were analysed with Jasco w32 software (Jasco). Deconvolution of the absorption spectra was done with Gaussian profile and Full Width at Half Maximum (FWHM). All peak heights of deconvoluted spectra were measured using a two-point base on either side of the peak. The normalised intensities for all peaks were calculated using the intensity of the reference band at 1024 cm^−1^, assigned to invariant CO stretching [12,29].

## 3. Results and Discussion

### 3.1. XRD Results

An X-ray diffractogram of pine wood samples, including the crystal lattice assignments, is shown in Figure 1. The diffraction pattern is similar to that of cellulose, which was characterised by two main peaks and a broad amorphous background band.

The lower angle peak was the result of the merging of the diffraction peaks at 2θ = 15° and 16.5° into a broader one, as also reported in the literature [23], where it is assigned to the [001] crystalline plane. The peak observed at 2θ = 22.4° was assigned to the [002] crystalline plane and was used for the calculation of the crystallinity index CI_XRD_.

**Figure 2:**
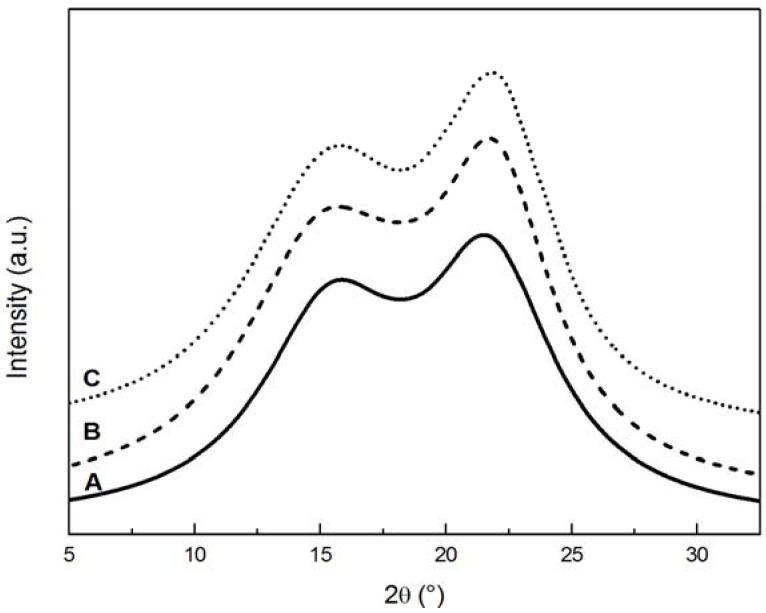
XRD diffractograms of coated wood samples. (**A**) control; (**B**) sample exposed to natural weathering; (**C**) sample artificially weathered.

The effect of natural and artificial weathering on the X-ray diffractograms of uncoated pine wood can be observed in Figure 1. The peak at 2θ = 22.4° became sharper and its height increased significantly while the height of the other peak at 15.5° remained substantially unchanged. As expected, the effect of weathering was less evident on coated samples, as shown in Figure 2, because of the protective action of the coating.

The changes in diffractograms were analysed in order to determine the crystallinity index (CI) and the thickness of crystallites, whose values are reported in Table 1. The values of CI_XRD_ agree with those reported by Andersson *et al.* [30] who found a crystallinity of wood varying from 24% to 31% for Scots pine wood not exposed to weather. In Table 1, ∆CI_XRD_ and ∆_thickness_ represent the percentage of variation in properties compared to the control specimen of the same group (coated and uncoated).

The increase of CI can be attributed to the degradation caused by weathering, which reduces the amorphous fractions of wood and, consequently, enriches the relative crystalline content, taking into account that less than one third of the wood polysaccharides are crystalline and the remaining wood constituent are hemicelluloses, pectic substances, or amorphous and para-crystalline regions of the cellulose fibrils. If those amorphous polysaccharides are degraded more than the crystalline cellulose, the overall crystallinity content is expected to increase. The apparent increase in the crystalline fraction of cellulose observed in the studied wood sample well agrees with that reported by Fackler *et al.* [20] and Salaita *et al.* [31] on wood subjected to fungal decay and weathering, respectively.

XRD measurements on samples naturally weathered for two years were also performed. The results were not significantly different from those obtained on one year weathered samples. This suggests that degradation of the amorphous phases could become a steady process when some of the species produced by degradation and which are insoluble in water may form a surface layer which protects the underlying wood from further degradation.

**Table 1:** Crystallinity index CI_XRD_ and crystallite thickness as obtained by XRD measurements on weathered wood.

Sample	CI_XRD_ (%)	∆CI_XRD_ (%)	Crystallite thickness (nm)	∆_thickness_ (%)
COATED				
control	22.1	–	3.10	–
naturally aged	27.4	24%	3.50	13%
artificially aged	28.1	27%	3.61	16%
UNCOATED				
control	33.1	–	3.11	–
naturally aged	46.3	40%	3.80	22%
artificially aged	63.1	90%	4.09	31%

The crystallite thicknesses, determined by applying the Scherrer formula (Table 1) for pine wood was 3.1 nm, in agreement with the literature [30]. Weathering resulted in an increase of the crystallite thickness from 3.11 to 3.84 nm for uncoated wood and from 3.10 to 3.61 nm for coated wood. This phenomenon has been also observed in the literature for heat-treated wood [19]. In this case, it can be assumed that a reduction of molecular weight of cellulose due to thermo-oxidative processes is associated with an increase of CI. Howell *et al.* [19] hypothesised that the apparent changes in CI and crystallite size may be due to a re-crystallization of the semicrystalline wood component after the removal of the amorphous fraction (lignin and hemicellulose). However, this process is difficult to observe with FT-IR because of the difficulty to separate the intensity bands related to hemicelluloses and semicrystalline cellulose.

### 3.2. FT-IR Results

**Figure 3:**
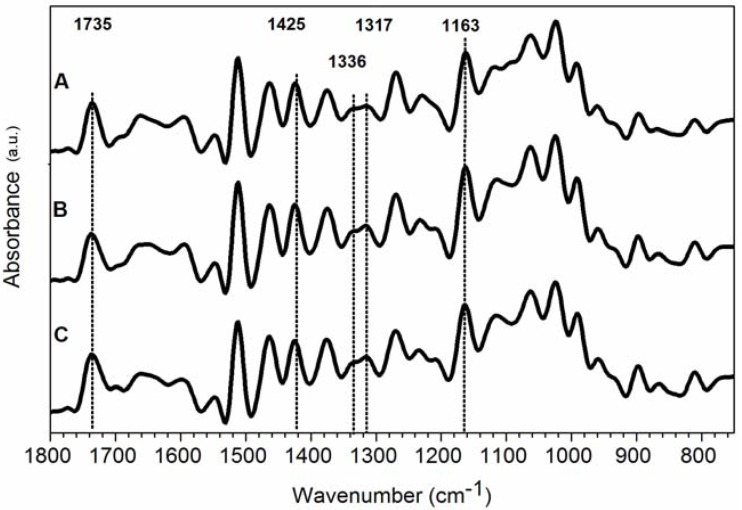
Deconvoluted Fourier transform infrared (FT-IR) spectra of coated wood samples. (**A**) control; (**B**) sample exposed to natural weathering; (**C**) sample artificially weathered.

**Figure 4:**
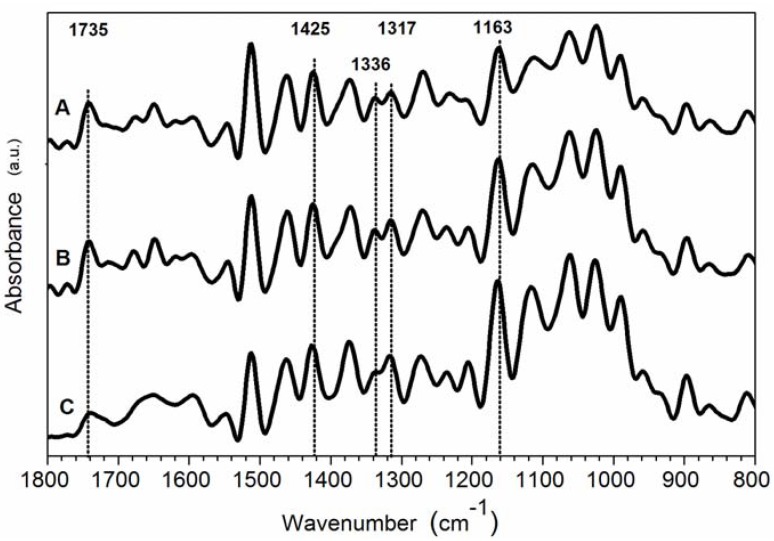
Deconvoluted FT-IR spectra of uncoated wood samples. (**A**) control; (**B**) sample exposed to natural weathering; (**C**) sample artificially weathered.

Since FTIR spectra of wood samples feature overlapping bands, all spectra were analysed after a deconvolution. Figure 3 and Figure 4 show FT-IR spectra in the fingerprint region between 1800 and 800 cm^−1^ for coated and uncoated pine wood samples, respectively, at different weathering conditions.

The assignments of characteristic IR bands to various components of wood are summarised in Table 2. The band at 1735 cm^−1^ was characteristic of an unconjugated carbonyl group typical of xylan and hemicelluloses. Lignin bands were found at 1595 cm^−1^ and 1512 cm^−1^ for C=C stretching of the aromatic ring, at 1463 cm^−1^ for CH_3_ bending and at 1269 cm^−1^ for CO stretching in lignin (guaiacyl) and hemicellulose. Finally, typical bands assigned to cellulose were located at 1425 cm^−1^ and 1375 cm^−1^ for CH_2_ and CH bending mode, respectively, at 1163 cm^−1^ and 897 cm^−1^ and at 1336 cm^−1^ for hydroxyl bending, and 1317 cm^−1^ for CH_2_ wagging, which distinguished between amorphous and crystallised I cellulose [20,29].

**Table 2:** Assignments of characteristic absorption IR bands of wood samples in fingerprint region.

Wavenumber (cm^−1^)	Functional group	Assignment
1735	C=O stretching in unconjugated ketones aldehydes and carboxyl	Xylan and hemicellulose
1595	C=C stretching of the aromatic ring	Lignin
1512	C=C stretching of the aromatic ring	Lignin
1463	Asymmetric bending in CH_3_	Lignin
1425	CH_2_ bending	Cellulose (crystallised I and amorphous)
1375	CH bending	Cellulose
1336	OH in plane bending	Cellulose (amorphous)
1317	CH_2_ wagging	Cellulose (crystallised I)
1269	CO stretching	Lignin and hemicellulose
1163	COC asym. bridge oxygen stretching	Cellulose
897	asym. Out of phase ring stretching	Cellulose

**Figure 5:**
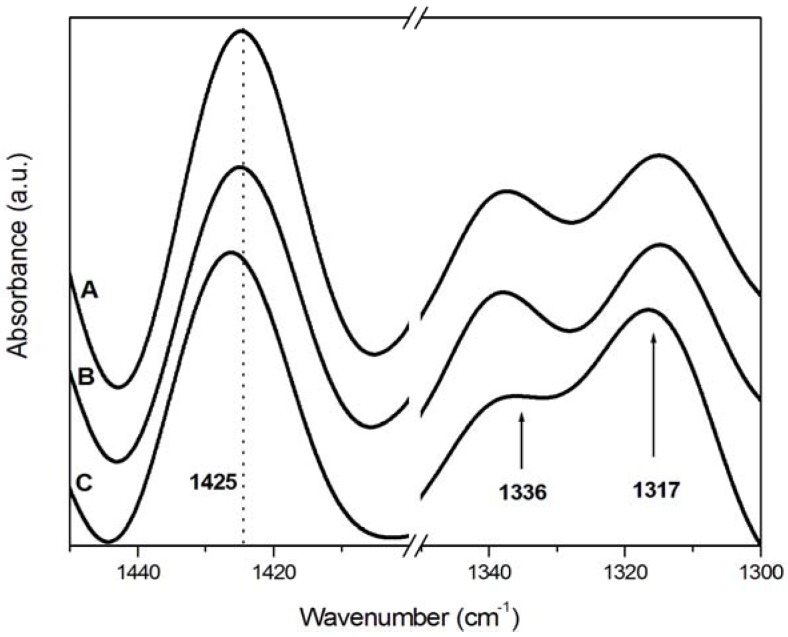
Deconvoluted FT-IR spectra of uncoated wood samples in the region 1300–1450 cm^−1^. (**A**) control; (**B**) sample exposed to natural weathering; (**C**) sample artificially weathered.

The changes in IR spectra due to weathering of uncoated samples are shown in Figure 4. The band at 1425 cm^−1^ slightly shifted to higher wavelength in aged samples (curves B and C in Figure 4 and Figure 5). According to Colom *et al.* [12], a shift of the band at 1425 cm^−1^ (assigned to amorphous and crystallised cellulose) to 1430 cm^−1^ (characteristic of crystallised cellulose), indicated that the amorphous area of the cellulosic component was more affected by the degradation process, or that partially degraded cellulose was capable of forming new and larger crystals, as also observed by XRD.

The band at 1163 cm^−1^ (Figure 4) slightly shifted to higher wavelengths and became narrower. Usually in crystallised cellulose, this band was located at 1163 cm^−1^ while in amorphous cellulose at 1156 cm^−1^. A qualitative decrease of amorphous cellulose content can be thus assumed in uncoated samples exposed to weathering [12,20].

After weathering the C=O stretching band at 1740–1720 cm^−1^ can increase, due to an oxidative degradation of lignin caused mainly by UV light [1]. Furthermore, the C=O stretching band at 1740–1720 cm^−1^ can decrease, due to degradation of acetyl groups [20,29]. Moreover, a shoulder around 1730 cm^−1^ can be due to oxidised cellulose and lignin [1,20,29]. No considerable changes in the absorbance at 1735 cm^−1^ were found for coated samples after ageing (Figure 3), suggesting no photo-oxidation of the wood surface or consistent deacetylation of hemicellulose. On the other hand, a significant decrease in the absorbance value at 1735 cm^−1^ (from 0.51 to 0.15) for uncoated wood after artificial ageing (curve C in Figure 4) is suggestive of a consistent degradation of acetyl groups promoted only by artificial treatment onto unprotected wood surfaces. A detailed analysis of carbonyl groups region for wood plastic composites subject to degradation was described by Fabiyi *et al.* [32,33]. Four different carbonyl groups were found in the region 1800–1680 cm^−1^, mainly as overlapped bands, assigned to γ-lactone (1800–1765 cm^−1^), ester (1745–1730 cm^−1^), hydrogen-bonded carboxylic acids (1725–1715 cm^−1^) and conjugated ketones (1700–1685 cm^−1^). They found an increasing concentration of both esters at 1735 cm^−1^ and carboxylic acid at 1715 cm^−1^ upon xenon-arc and UVA weathering, while with longer exposure time, these concentrations began to decrease [32]. In our study, for coated samples, the region between 1740 cm^−1^ and 1710 cm^−1^ (esters and carboxylic acids) remained almost similar after ageing. The uncoated samples after natural ageing showed a very slight decrease of the ester signal and a small signal at 1714 cm^−1^ due to carboxylic acid. After artificial ageing, only a shoulder at 1714 cm^−1^ was observed in the uncoated samples confirming that, after a stronger ageing, both ester and carboxylic acid underwent remarkable degradation.

A quantitative analysis was carried out, focused on the changes of the intensity of the absorptions at 1735, 1512, 1463, 1425, 1375, 1336, 1317, 1269, 1163, and 897 cm^−1^. All the intensities of IR bands were normalised to the intensity of the 1024 cm^−1^ band in the deconvoluted spectra. Even if the chosen band, due to both lignin and carbohydrate C–O stretching, did not remain completely constant in all spectra, it represents one of the less variable bands during the ageing. All data are summarised in Table 3.

**Table 3:** FT-IR absorbances average values of wood samples. All values are normalised to 1024 cm^−1^.

	Sample	Band frequency (cm^−1^)									
		1735	1512	1463	1425	1375	1336	1317	1269	1163	897
Absorbance (a.u.)	COATED										
	control	0.52	1.06	0.59	0.44	0.34	0.11	0.14	0.46	0.60	0.21
	naturally aged	0.43	0.99	0.59	0.46	0.38	0.15	0.19	0.41	0.64	0.24
	artificially aged	0.61	1.06	0.66	0.47	0.44	0.16	0.20	0.38	0.65	0.29
	UNCOATED										
	control	0.51	1.27	0.72	0.62	0.45	0.28	0.32	0.43	0.67	0.31
	naturally aged	0.40	0.80	0.50	0.45	0.37	0.25	0.29	0.27	0.70	0.27
	artificially aged	0.15	0.61	0.39	0.39	0.38	0.22	0.30	0.24	0.80	0.30

Characteristic lignin bands at 1512, 1465 and 1269 cm^−1^ show a similar trend. In fact, all these three bands did not change significantly after weathering of coated wood while they showed a significant decrease in absorbance values for uncoated wood samples. An absorbance reduction of 0.47; 0.22 and 0.16 in naturally weathered samples and a reduction of 0.66; 0.33; 0.19 in artificially weathered samples has been found in uncoated samples due to a severe degradation of lignin, whereas lignin degradation seems mostly preserved by a protective layer.

Even if absorbance values considered individually give useful information on wood degradation during the ageing process, additional information has been obtained from height ratios calculated between them. The height ratios I_1375_/I_1512_ and I_1317_/I_1336_ are reported in Table 4.

**Table 4:** Absorbance ratios of wood samples with the indication of percentage variation in properties between aged and control samples.

Sample	CI_FTIR_ I_1375_/I_1512_	∆CI_FTIR_ (%)	I_1317_/I_1336_	∆ (%)
COATED				
control	0.32	–	1.27	–
naturally aged	0.38	19	1.27	0
artificially aged	0.42	31	1.25	−1
UNCOATED				
control	0.35	–	1.14	–
naturally aged	0.47	34	1.16	2
artificially aged	0.63	80	1.36	19

The absorbance ratio I_1375_/I_1512_ can be used as the crystallinity index (CI_FTIR_) of wood considering that the peak at 1375 cm^−1^ was characteristic of cellulose and that at 1512 cm^−1^ of lignin [20]. This ratio increased for the coated wood sample after weathering, thereby indicating the degradation of lignin when wood was protected by a coating layer. In accordance to XRD results discussed above, a higher CI was found for uncoated wood, especially when artificial ageing was used, where a significant degradation of lignin was observed.

The absorbance ratio I_1317_/I_1336_ provided additional information concerning the difference in the degradation process of amorphous and crystalline cellulose. Since the 1317 cm^−1^ and 1336 cm^−1^ bands were related to the contents in crystallised I and amorphous cellulose, respectively, as reported in Table 1, an increase in the ratio indicated an increase in crystallinity [12]. The ratio remained almost constant in all samples except for the uncoated artificially aged sample where a decrease was found. This indicated that the degradation of the amorphous cellulose component became significant only if wood was exposed to the artificial weathering conditions.

Therefore, the FTIR results confirmed the reduction of the lignin and hemicellulose component of wood samples due to weathering as also found by XRD analysis. Moreover, the FTIR technique was able to distinguish between the behaviour of lignin and hemicellulose, suggesting that hemicellulose underwent to a severe degradation only in the artificial ageing of uncoated wood samples.

**Figure 6:**
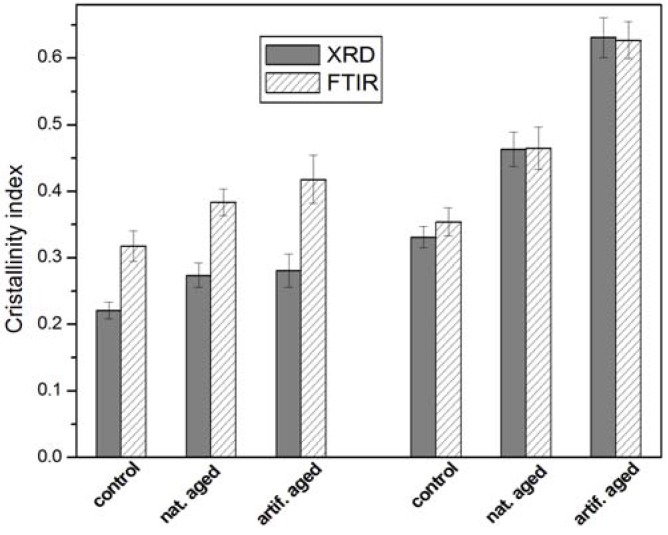
Crystallinity index (CI) of wood cellulose determined by XRD and FT-IR.

Assuming CI_FTIR_ as the I_1375_/I_1512_ absorbance ratio, the results obtained with the two techniques did not provide the same values of CI in absolute terms. However, as reported in Figure 6, the results obtained by the two techniques presented the same growth trend with the weathering. Therefore, it was possible to correlate the CI obtained by XRD (CI_XRD_) with that one obtained by FT-IR spectroscopy (CI_FTIR_).

A correlation between CI_FTIR_ (I_1375_/I_1512_) and CI_XRD_ values gave a high correlation coefficient (0.94). The CI obtained from FT-IR (CI_FTIR_) showed to correlate well with the corresponding CI obtained by X-ray diffraction (CI_XRD_).

It should be highlighted that the paint was applied on a rough surface and the ageing was responsible for the changing of both the coating thickness and colour. Therefore, it cannot be excluded that the complete removal of the coating may be associated with the removal of a very small amount of wood, in the order of tens of microns, at the interface between coating and wood. This may lead to an underestimation of the values of the crystallinity index. Despite this, the significant differences found in the CI values among control and weathered samples, obtained by XRD and FTIR, demonstrate how both the techniques are sensitive to small changes due to weathering. This is further proof of the reliability of the proposed spectroscopic approach to monitor the degradation of wood.

## 4. Conclusions

This work presents a spectroscopic approach, based on XRD and FTIR, to monitor the degradation of the different amorphous components of wood during weathering.

The observed increase in the CI of wood cellulose is due to the enrichment of the relative crystalline content at the expense of the amorphous fractions of wood. The results obtained by the two techniques can be correlated, providing complementary information about the degradation intensity and mechanisms.

The techniques applied in this paper are not routinely used for monitoring wood degradation. However, their reliability for evaluating wood degradation and, hence, the protective efficacy of wood coatings has been proved. These results can be easily applied to the field of wood coatings.

Finally, the presented work is the first part of a complete characterisation of solid wood during weathering. Weathering experiments on longer periods are in progress. Further research will be devoted to refine the proposed method by applying it on new coating formulations and on wood exposed to weathering for different periods.
